# Real world usage characteristics of a novel mobile health self-monitoring device: Results from the Scanadu Consumer Health Outcomes (SCOUT) Study

**DOI:** 10.1371/journal.pone.0215468

**Published:** 2019-04-16

**Authors:** Jill Waalen, Melissa Peters, Daya Ranamukhaarachchi, Jenny Li, Gail Ebner, Julia Senkowsky, Eric J. Topol, Steven R. Steinhubl

**Affiliations:** 1 Scripps Research Translational Institute, La Jolla, California, United States of America; 2 Scanadu, Sunnyvale, California, United States of America; 3 Wave Research Center, Los Angeles, California, United States of America; Texas A&M University, UNITED STATES

## Abstract

A wide range of personal wireless health-related sensor devices are being developed with hope of improving health management. Factors related to effective user engagement, however, are not well-known. We sought to identify factors associated with consistent long-term use of the Scanadu Scout multi-parameter vital sign monitor among individuals who invested in the device through a crowd-funding campaign. Email invitations to join the study were sent to 4525 crowd-funding participants from the US. Those completing a baseline survey were sent a device with follow-up surveys at 3, 12, and 18 months. Of 3872 participants receiving a device, 3473 used it during Week 1, decreasing to 1633 (47 percent) in Week 2. Median time from first use of the device to last use was 17 weeks (IQR: 5–51 weeks) and median uses per week was 1.0 (IQR: 0.6–2.0). Consistent long-term use (defined as remaining in the study at least 26 weeks with at least 3 recordings per week during at least 80% of weeks) was associated with older age, not having children in the household, and frequent use of other medical devices. In the subset of participants answering the 12-month survey (n = 1222), consistent long-term users were more likely to consider the device easy to use and to share results with a healthcare provider. Thirty percent of this subset overall reported improved diet or exercise habits and 25 percent considered medication changes in response to device results. The study shows that even among investors in a device, frequency of device usage fell off rapidly. Understanding how to improve the value of information from personal health-related sensors will be critical to their successful implementation in care.

## Introduction

The potential for mobile health technology (mHealth) to transform approaches to health on both a personal and population level has long been recognized.[1,2] On the personal health level, mHealth technology continues to produce increasing numbers of devices that are ever smaller and more wearable and capable of measuring increasing numbers of physiologic parameters. Smartphones alone or linked to a range of wireless sensors can routinely monitor activity in the form of steps, heart rate and rhythms, sleep duration and quality, temperature, oxygen saturation and blood pressure. Smart watches and other wearables are making the monitoring simpler, continuous and less obtrusive.

Understanding of the real-world use and health-related effects of consumer-focused digital health technologies, however, has not kept pace. Often developers of these technologies assume that enthusiasm for potential health benefits will translate into consistent use. However, qualitative research has found that individuals may perceive personal health data tracking as work that also has an emotional burden.[3] While too few health-related apps and devices have reported long-term usage characteristics, those that have suggest very rapid drop off of the vast majority of users—90% or more. [4,5]

Similar rapid attrition has been found in several well-designed, smartphone-based research programs. For example, in the MyHeart Counts Cardiovascular Study using an Apple ResearchKit app to track the activity of 40,000 participants via smartphone, less than 50% of subjects provided two consecutive days of activity data, with less than 10% completing all 7 days.[6] A similar lack of extended engagement has been described with other app-based research programs including the Asthma Mobile Health Study, the mPower Study, and others.[7–9] The results have led commentators to note that learning how to optimize digital engagement is a “cutting-edge scientific challenge” that will be salient to the incorporation of these technologies into routine health management.[10]

**Fig 1:**
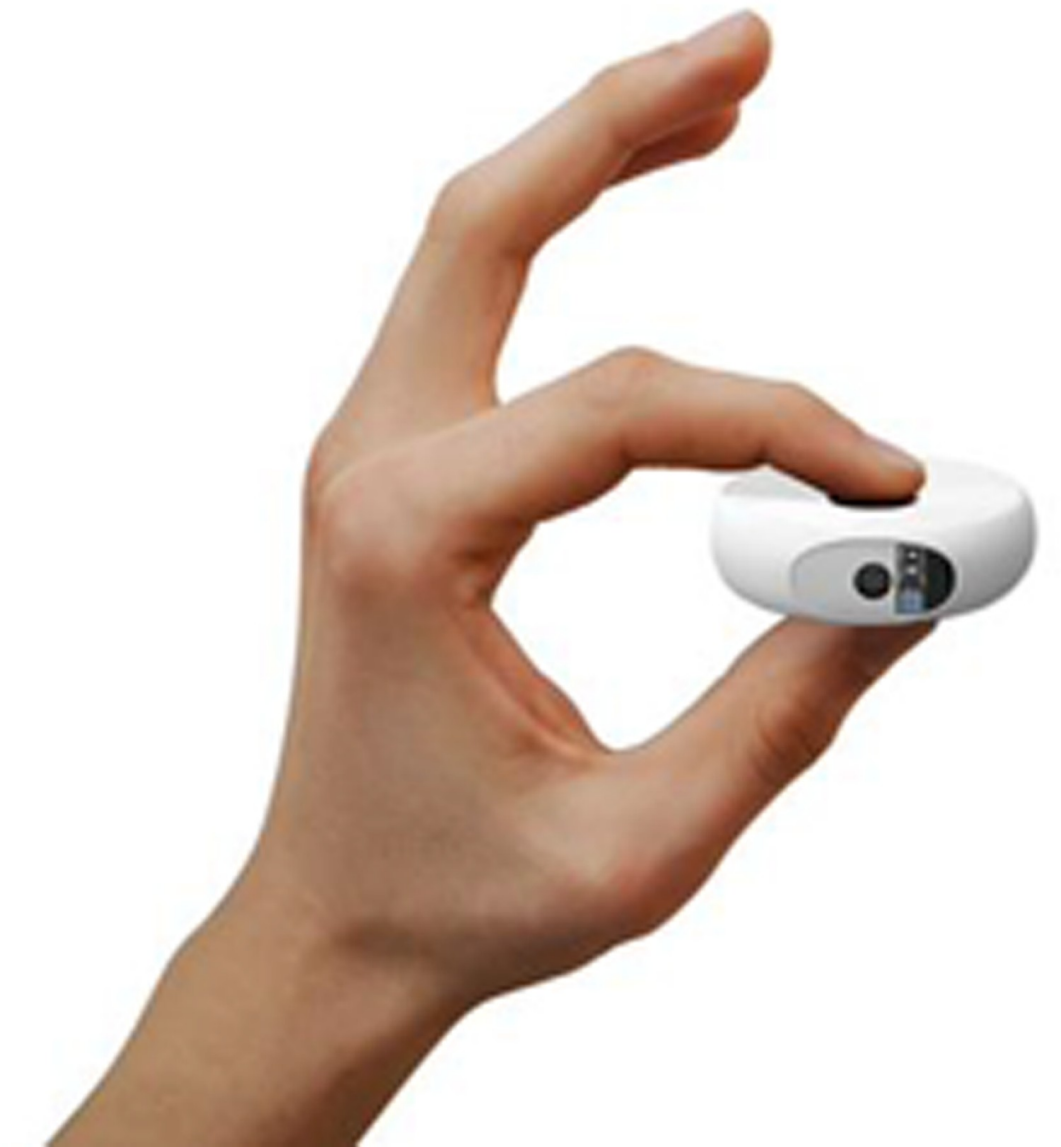
Scanadu scout device. The Scanadu Scout device records physiological measurements when held to the forehead. (Source: Scanadu with permission).

In the current study we assessed the real-world use of the Scanadu Scout, a pocket-sized device that, when held to the forehead, was designed to measure four physiologic parameters: heart rate, blood pressure, body surface temperature and oxygen saturation through pulse oximetry (Fig 1). The device was offered for use to individuals who chose to participate in a crowdfunding campaign to support the development of the device, through an IRB-approved protocol involving completion of several online surveys and monitoring of device usage via time-stamped recording. We describe usage patterns and characteristics associated with consistent long-term use of the device over 18 months among this cohort.

## Methods and materials

### Study population

Contributors to the crowdfunding campaign for the Scanadu Scout conducted during May-July, 2013 and living in the United States (4525 individuals) received an invitation to participate in a study involving use of the device for 18 months beginning in January 2015. The invitation included a link to the online study platform, which contained detailed information about the study’s purpose to evaluate usage characteristics, the methods to be used, and the informed consent document. Potential participants were explicitly informed that the device was not yet validated to be accurate and was not FDA approved and that therefore no healthcare decisions should be made based on the results of the device. Consent was obtained online with digital signature and in signing, participants agreed to restrict the use of the device to personal use only, and not allow family members or friends to use it. After signing the Informed Consent, the participant was asked to complete a baseline survey through the study platform and those completing the survey were sent a Scanadu Scout device. The study was limited to participants age 18 years or older. There were no other exclusion criteria. The study was approved by the Scripps Institutional Review Board and was carried out in accordance with the approved protocol.

### Survey instruments

All surveys were administered online on a secure website. The baseline survey included questions about participants’ demographics including medical background, attitudes about sharing the device data with health care providers, current use of related technologies, current health status and sources of health information, as well as questions about adoption of innovations and health-related quality of life (Table 1).

**Table 1:** Study assessments.

		Baseline	3 Months	12 Months	18 Months
**Participant Characteristics**	Demographics	x			
	Technology Utilization	x			
	Diffusion of Innovations[11]	x			
**Health Outcomes**	Health-Related Quality of Life[12]	x	x	x	x
	Medication		x	x	x
**Lifestyle Outcomes**	Diet changes[13]		x	x	x
	Exercise changes[14]		x	x	x
	Health Communication		x	x	x
**Perception of Device**	Usability and Satisfaction		x	x	x
	Device Design/Labeling		x	x	x

At 3, 12, and 18 months, all participants were prompted by e-mail to complete a follow-up survey accessed with a link to the secure website. Surveys included questions on medication changes, lifestyle changes (diet and exercise), sharing of data with health care providers, and perceptions of usability of the device, changes in diet and exercise, and the same questions on health-related quality of life as the baseline survey (Table 1).

Reminder e-mails were sent to participants not completing a follow-up survey within 7 days. For participants completing the last follow-up survey, the study ended at 18 months from enrollment. All other participants were considered withdrawn at the time of the last completed follow-up survey. At study completion the Scout’s functionality was disabled remotely by Scanadu.

### Device usage/physiologic measurements

**Fig 2:**
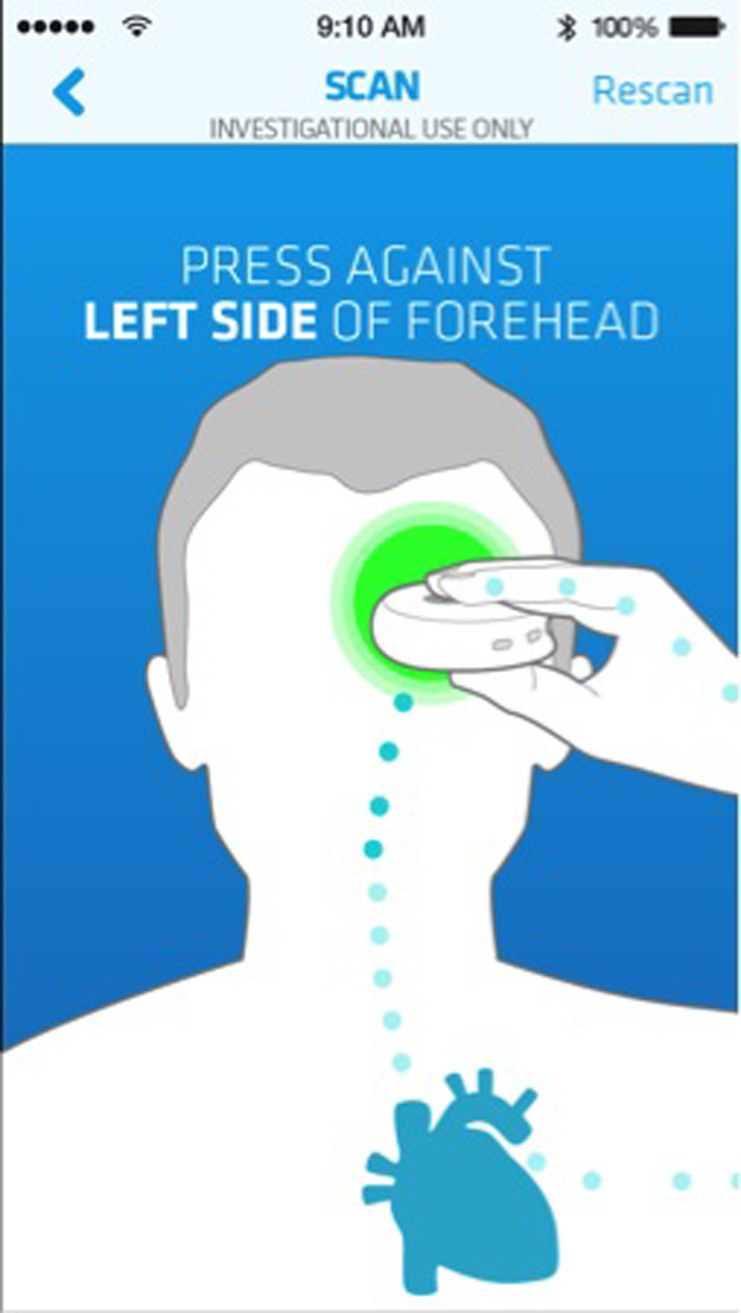
Scanadu scout smartphone application. Screenshots illustrating device placement from downloaded smartphone application. (Source: Scanadu with permission).

Upon receipt of the device, participants were instructed to download its associated application from the iOS or Android App Stores. The application was activated by handshake with the participant’s device via Bluetooth connection. Instructions for device placement and readings from the device were displayed on the smartphone (Fig 2). Device usage data and physiologic measurements stored in the app were passively collected by Scanadu and stored for analysis. Participants were instructed to use the device as much as they would like, but not to make any medical decisions based on the measurements. “For Investigational Use” was displayed on every screen. Device usage data was truncated for analysis at the time of study withdrawal or at the end of the study.

### Statistical considerations

As an observational, exploratory study, sample size was determined by the size of the crowdfunding cohort and their willingness to participate. Descriptive statistics including all items from the baseline questionnaire were calculated. Primary endpoints included frequency of device usage as well as changes measured in the 3 follow-up surveys in health-related quality of life; lifestyle outcomes (medications, diet, exercise and health communication); and perceptions of the Scanadu Scout (satisfaction with technology and device design/labelling assessments). Device usage was characterized by weeks of use (i.e. any device use in that one week period) and average number of uses per week. Responses to the surveys by individuals with the highest level of use (“consistent long-term users” post hoc defined as an average of 3 recordings per week and use for at least 80% of weeks and remaining in the study for at least 26 weeks) were compared with the rest of the cohort using the t-test for continuous variables and Fisher’s exact test or Chi square test for categorical variables. All statistical tests were two-sided with p< 0.05 considered statistically significant. Multivariable logistic regression was used to establish adjusted associations of frequent device use. Consistent with the exploratory nature of the study, no adjustments were made for multiple comparisons.

## Results

### Baseline characteristics

**Fig 3:**
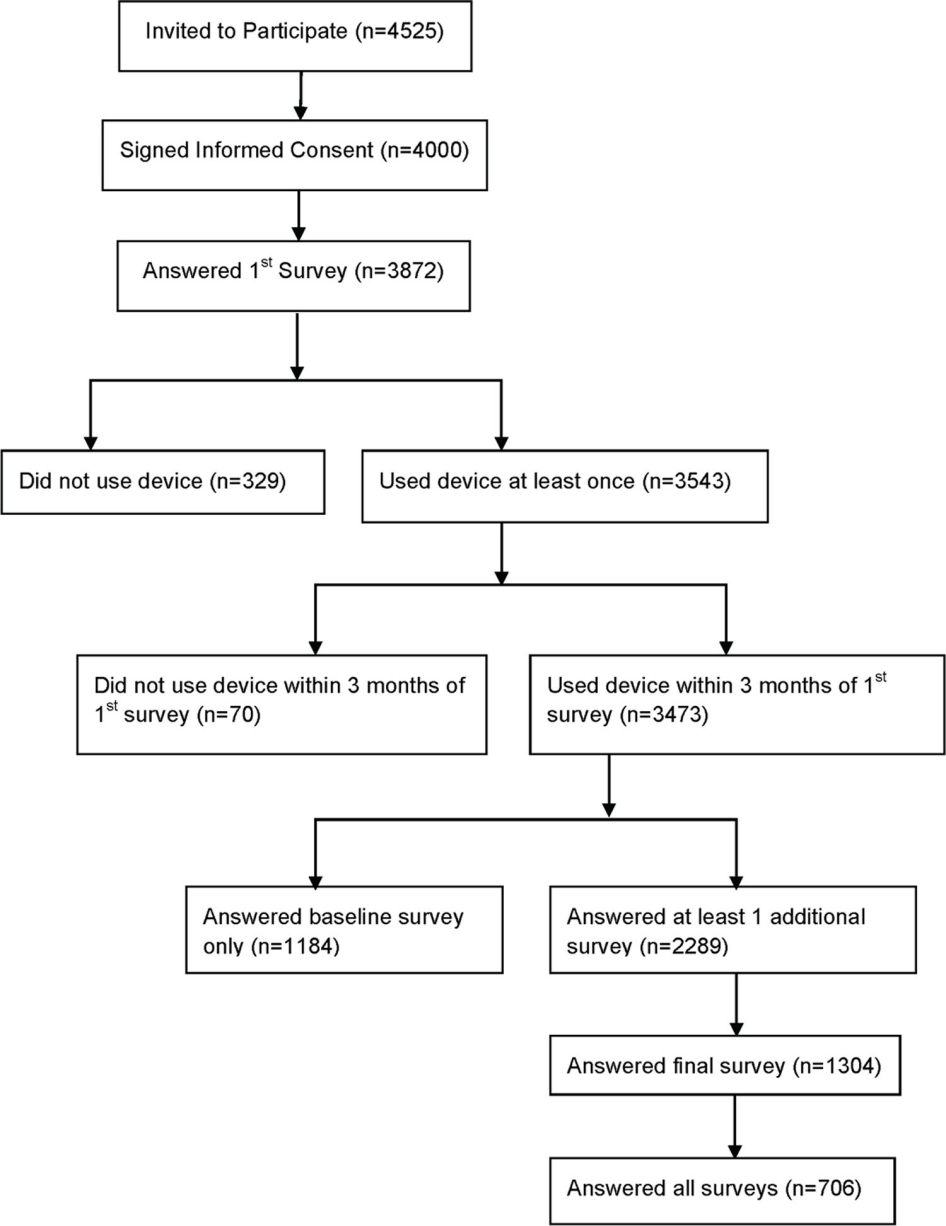
Participant flow chart.

Of the 4525 individuals invited to participate, 4000 subjects signed the informed consent and 3872 completed the baseline survey (Fig 3). Participants’ mean (standard deviation) age was 47 (13) years; 84% were men, most were married or had a domestic partner (66%) and had a bachelor’s or graduate degree (79%). Overall, 45% reported having at least one current health problem (Table 2). The cohort reported high levels of medical device use, with more than 75% of participants using a device (which included scales, thermometers and home blood pressure monitors) at least once a week. Use of “smart wellness” technologies was also prevalent, with more than 50% reporting consistent use of an activity tracker and 43% using mobile health apps (Table 2). The majority agreed that mobile devices were consistent with their approach to health (87%) and could help them stay healthier (70%) (S1 Table). Compared with men, women in the cohort were slightly older (mean age 51 years for women vs. 47 years for men) and more likely to report limitations of activity due to health, among other statistically significant differences (Tables 2 and S1).

**Table 2:** Baseline characteristics of all respondents to baseline survey, overall and by sex.

Characteristic	Overall (n = 3872)	Men (n = 3249)	Women (n = 623)	p*
	Mean (SD)	Mean (SD)	Mean (SD)	
**Age (years)**	47.4 (12.8)	46.8 (12.6)	50.5 (13.5)	< .001
**Age Range (years)**	20–89	20–89	21–88	
**Household Composition**	**n (%)**	**n (%)**	**n (%)**	
Single	1041 (26.9)	825 (25.4)	216 (34.7)	< .0001
Married/Domestic Partner	2544 (65.7)	2190 (67.4)	354 (56.8)	< .0001
Children	1380 (35.6)	1226 (37.7)	154 (24.7)	< .0001
Parents	230 (5.9)	190 (5.8)	40 (6.4)	0.52
**Level of Education**				
<12 years	9 (0.2)	7 (0.2)	2 (0.3)	0.09
High School/GED	84 (2.2)	72 (2.2)	12 (2.0)	
Some College	538 (14.0)	471 (14.6)	67 (10.9)	
Associate Degree	163 (4.3)	128 (4.0)	35 (5.7)	
Bachelor's Degree	1444 (37.6)	1205 (37.5)	239 (38.9)	
Graduate Degree	1593 (41.6)	1334 (41.5)	259 (42.2)	
**Medical Background**				
No	2803 (72.4)	2386 (73.4)	417 (66.9)	< .0001
Some	611 (15.8)	515 (15.9)	96 (15.4)	
Professional	458 (11.8)	348 (10.7)	110 (17.7)	
**Frequency of Use of Medical Devices**				
Do not use	860 (22.3)	702 (21.7)	158 (25.4)	0.03
1–6 times a week	2008 (52.0)	1679 (51.8)	329 (53.0)	
Once daily	874 (22.6)	755 (23.3)	119 (19.2)	
Multiple times a day	122 (3.2)	107 (3.3)	15 (2.4)	
**Use of "Smart" Wellness Technology**				
Activity Tracker	2068 (53.4)	1747 (53.8)	321 (51.5)	0.30
Smart Scale	1174 (30.3)	1049 (32.3)	125 (20.1)	< .0001
Smart Watch	923 (23.8)	843 (26.0)	80 (12.8)	< .0001
Sleep Tracker	1085 (28.0)	926 (28.5)	159 (25.5)	0.13
Mobile Health App	1668 (43.1)	1438 (44.3)	230 (36.9)	0.0007
**Early Adopter of Medical Technology?**				
Agree	2611 (68.0)	2228 (69.1)	383 (62.4)	< .0001
Neutral	927 (24.1)	781 (24.2)	146 (23.8)	
Disagree	302 (7.9)	217 (6.7)	85 (13.8)	
**Current Health Problem**	1725 (44.7)	1397 (43.1)	328 (53.1)	< .0001
**Limitation of Moderate Activities**				
No, not limited at all	3178 (82.6)	2715 (84.0)	463 (75.2)	< .0001
Yes, limited a little	480 (12.5)	382 (11.8)	98 (15.9)	
Yes, limited a lot	192 (5.0)	137 (4.2)	55 (8.9)	

*p for difference between men and women by t-test (age) Fisher’s exact test or Chi square (categorical variables)

### Device usage

**Fig 4:**
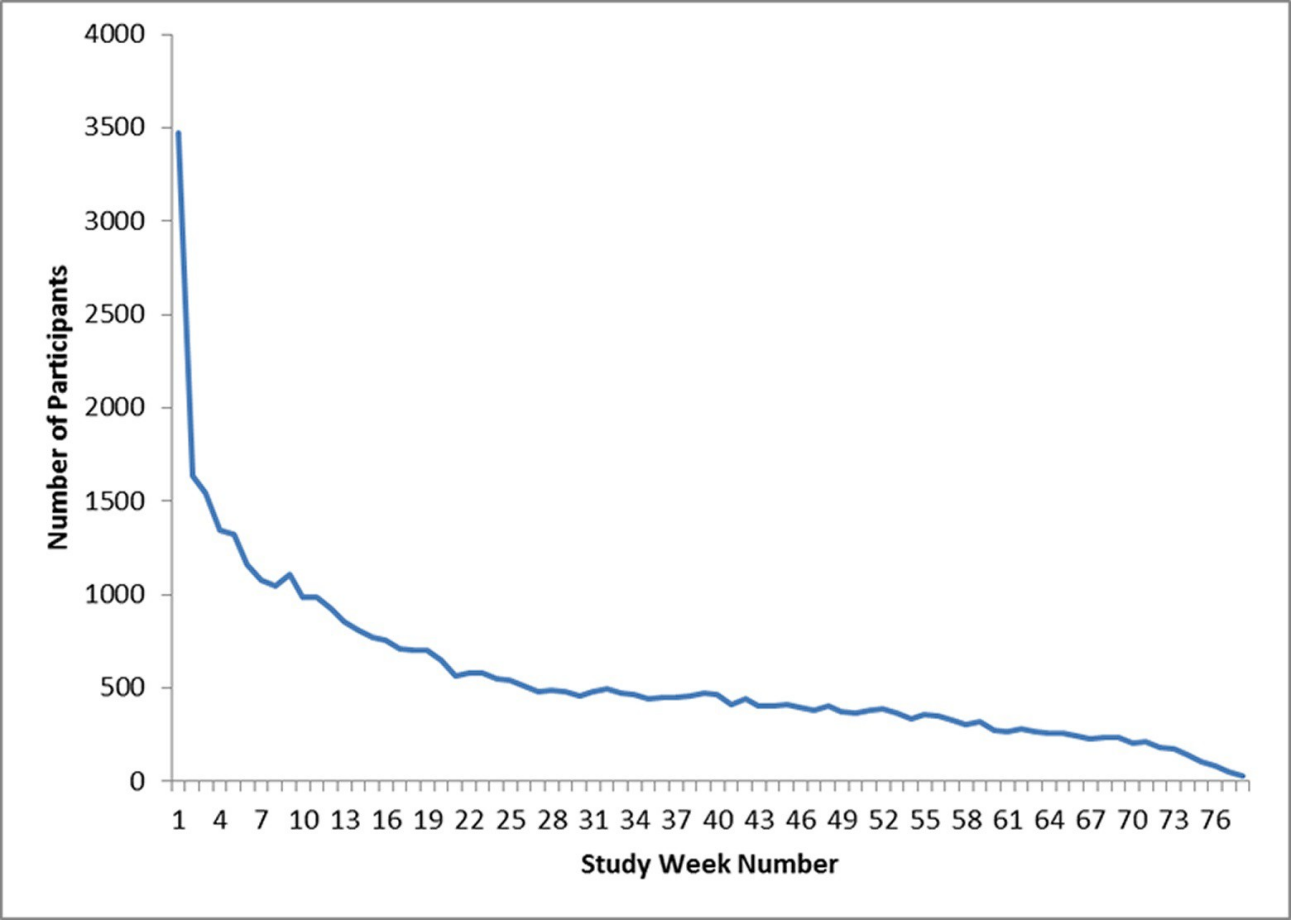
Total number of participants using the device over time (by study week number). Week 1 on the x-axis refers to the participant’s first week of using the device. The y-axis indicates the number of participants recordings at least one use of the device during each week they were in the study.

**Fig 5:**
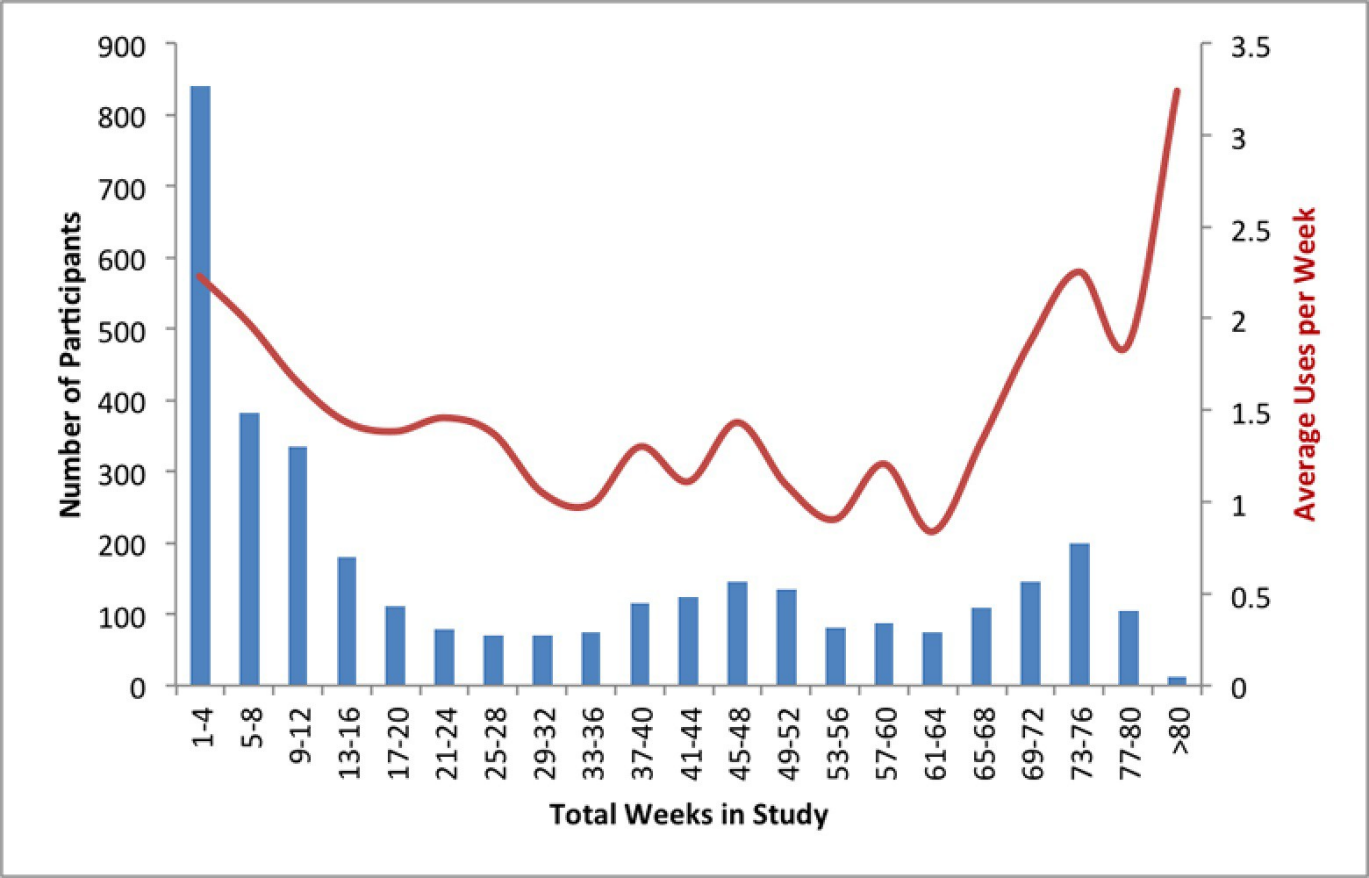
Amount of device usage by total time in study. X-axis indicates total time in study from device recording to last device recording. Y-axis on left indicates number of participants who remained in the study for the given total time intervals. Y-axis on the right average number of times device per week by the participants in each of the time intervals.

A total of 3543 participants who completed the baseline survey downloaded the app and used the device at least once, with 3473 recording their first use within 3 months of completing the first survey (Fig 3). The number of participants using the device at least once in the second week dropped to 1633 (47.0%) (Fig 4). Median time between first use of the device and last use was 17 weeks (IQR: 5–51 weeks) (Fig 5). Median number of uses per week in the study was 1.0 (IQR: 0.6–2.0). Those participating for 1 month or less averaged the most uses per week use, with 26.5% averaging more than 3 uses per week compared with 10% of participants in the study for more than 13 weeks (S2 Table and Fig 5). Three hundred thirty three participants (9.6%) recorded only 1 use of the device during the study.

### Baseline characteristics associated with frequent device usage

Comparing baseline characteristics of the consistent long-term users of the device—participants who were in the trial for at least 26 weeks with recordings during 80% of those weeks and averaging 3 or more uses per week, n = 93 (2.7% of the study population)—with the rest of the participants, consistent device users were statistically significantly older (mean age 56 years vs. 47 years) and more likely to live in a household without children (74% vs. 64%), use other medical devices at least once daily (45% vs. 26%), and report at least one current medical problem (54% vs. 45%) (Table 3). There were no differences in measures of health-related quality of life. In a multivariable logistic regression model of the association with frequent device uses which included all 4 variables, current medical problem was no longer statistically significant (S3 Table).

**Table 3:** Baseline characteristics of participants with linked device use by frequency of device use.

Characteristic	Consistent Long-term Users n = 93	All others n = 3380	p*
	**Mean (SD)**	**Mean (SD)**	
**Age**	56.4 (12.8)	46.9 (12.5)	< .0001
	**n (%)**	**n (%)**	
**Sex (% Men)**	83 (89.3)	2847 (84.2)	0.19
**Household Composition**			
Single	26 (28.0)	904 (26.86)	0.79
Married/Domestic Partner	63 (67.7)	2232 (66.0)	0.73
Children	15 (26.1)	1235 (36.5)	< .0001
Parents	5 (4.5)	201 (6.0)	0.82
**Level of Education**			
<12 years	0	9 (0.3)	0.02
High School/GED	6 (6.5)	68 (2.0)	
Some College	16 (17.4)	470 (14.0)	
Associate Degree	7 (7.6)	143 (4.3)	
Bachelor's Degree	26 (28.3)	1275 (38.1)	
Graduate Degree	37 (40.2)	1382 (41.3)	
**Medical Background**			
No	116 (74.8)	2399 (72.5)	0.45
Some	23 (14.9)	524 (15.8)	
Professional	16 (10.3)	395 (11.9)	
**Frequency of Use of Medical Devices**			
Do not use	14 (15.1)	731 (21.8)	< .0001
1–6 times a week	37 (39.8)	1776 (52.6)	
Once daily	33 (35.5)	766 (22.8)	
Multiple times a day	9 (9.7)	101 (3.0)	
**"Smart" wellness technology?**			
Activity Tracker	51 (54.8)	1835 (54.3)	0.92
Smart Scale	27 (29.0)	1051 (31.1)	0.67
Smart Watch	14 (15.1)	821 (24.3)	0.04
Sleep Tracker	29 (31.2)	978 (28.9)	0.64
Mobile Health App	41 (44.1)	148 (44.0)	0.99
None	28 (30.1)	928 (27.5)	0.57
**Early Adopter of Medical Technology?**			
Agree	108 (70.1)	2364 (68.7)	0.47
Neutral	20 (21.5)	796 (23.7)	
Disagree	9 (9.7)	251 (7.6)	
**Self-rated Health**			
**Currently Managing Personal Health Condition**	84 (54.2)	1474 (44.6)	0.02
**Limitation of Moderate Activities**			
No, not limited at all	76 (81.7)	2789 (82.9)	0.95
Yes, limited a little	12 (12.9)	409 (12.2)	
Yes, limited a lot	5 (5.4)	165 (4.9)	

*p for difference between sustained regular device users and all others by t-test (age) or Chi square (categorical variables)

### Follow-up surveys

Follow-up surveys answered by a subset of participants showed that at 12 months the majority of participants reported satisfaction with device use, with approximately 80% reporting the device was easy to use and 87% stating they would be willing to participate in a study testing the device again (Table 4). Overall, 28% of participants reported making changes to their diet and 34% made changes to their exercise routine. Despite continuous recommendations to the contrary, 26% of users reported making medication or supplement changes based on device results, 60% of whom reported consulting a doctor or other health care provider about the change (Table 4). However, there were no adverse events reported by any of the users in association with the medication changes. At 12 months, consistent long-term users of the device were statistically significantly more likely than the rest of the cohort to report sharing results from the device with their doctors or other healthcare provider (59% vs. 29%), that the device was easy to use (94% vs. 80%), that they were able to complete the scan to obtain measurements, that they would recommend the device to family and friends and that they would be likely to participate in a study of the device again (Table 4). When included in a logistic regression model controlling for the statistically significant baseline characteristics, ease of use and sharing results with healthcare providers remained independent predictors of consistent long-term use (S3 Table).

**Table 4:** Attitudes and behaviors of participants answering surveys at 12 months by amount of device use.

Question	Overall n = 1222*	Consistent Long-term Users n = 70	All others n = 1152	p**
	n (%)	n (%)	n (%)	
**Shared device results with physician or healthcare provider**	364 (30.1)	36 (52.9)	328 (28.8)	< .0001
**Thought about using device results for decisions on medications**	349 (28.6)	19 (27.1)	330 (28.7)	0.78
**Made changes to medications or supplements**				
No	903 (74.1)	52 (75.4)	851 (74.0)	0.62
Non-prescription medications	47 (3.9)	1 (1.5)	46 (4.0)	
Supplements	160 (13.1)	11 (15.9)	149 (13.0)	
Prescription medications	109 (8.9)	5 (7.3)	104 (9.0)	
**Consulted with a healthcare provider about medication or supplement change**	190/316 (60.1)	11/17 (64.7)	179/299 (59.9)	0.69
**Made changes to typical diet**	337 (27.8)	18 (26.1)	319 (27.9)	0.75
**Made changes to exercise routine**	410 (33.8)	28 (40.6)	382 (33.4)	0.22
**Would recommend device to family or friends**	956 (79.5)	61 (89.7)	895 (78.9)	0.03
**Agree with the following statements:**				
Device was easy to use	958 (81.4)	63 (94.0)	895 (80.6)	0.006
Able to open device and successful connect to app	1121 (95.3)	67 (95.1)	1054 (95.1)	0.20
Able to complete scan and obtain measurements	942 (80.0)	63 (92.7)	879 (79.3)	0.007
Using the device does not require too much of my time	943 (80.2)	62 (91.2)	881 (79.5)	0.02
The device is not distracting	1044 (89.2)	63 (94.0)	981 (88.9)	0.19
The device does not interrupt my daily activities	1044 (89.2)	61 (91.0)	893 (80.8)	0.04
I would be willing to participate in a study testing the device again	1019 (87.0)	65 (95.6)	954 (86.4)	0.03
**Self-rated Health**				
Limitation of Moderate Activities	240 (19.6)	17 (24.3)	223 (19.4)	0.31

*Denominators differ by question due to variable response rate.

**Chi square test

Participants who responded to all three of the follow-up surveys (n = 706) showed a marked change in the perception of the amount of time required to use the device and the distraction it caused, with approximately 60% reporting the device “interrupted my daily activities” and “required too much of my time” at 3 months decreasing to less than 20% at 12 and 18 months (S5 Table). Percent reporting diet and exercise changes increased significantly between 3 months and 12 months, plateauing at 30% and 36% at 18 months respectively.

## Discussion

Among a cohort of individuals self-identified as having very high interest in self-monitoring and digital health technologies in general, and the Scanadu Scout in particular, with high levels of interest and confidence in using devices for health-related purposes, we found an overall surprisingly low actual device usage, with the number of participants using the device dropping by 46% by the second week of the trial and less than 3% of participants overall remaining consistent users over the 18 month duration of the trial. In context, the participant cohort might be considered an outlier in the sense that they had even invested funds in the technology, which makes the findings noteworthy.

Using frequency of device use to indicate level of engagement among participants with any recorded data, we found individuals who used the device the most frequently over the greatest amount of time were older, more likely to live in households without children, and used other medical devices more frequently compared with other participants.

The results of this study, as well as multiple others of various digital health technologies, suggest that simply providing apps or devices that allow individuals to monitor their health, support wellness behaviors, or contribute to research programs, is inadequate to promote consistent long-term use for the majority of individuals. Although the current study was observational, and not designed to prospectively address low device usage, there are findings that support a major barrier to engagement is a lack of time, or stated another way, of inadequate value to an individual to dedicate their time to technology usage. The characteristics of the most frequent users of the Scanadu Scout—particularly being less likely to have dependents at home, a factor having one of the largest effects among baseline predictors in the study—are consistent with this. The finding underscores the importance of using proven behavior change approaches to promote use of digital health devices. Interest in the device, even to the extent of investing money in it, is not enough to ensure consistent use.

Results of the follow-up surveys completed by a subset of participants offer some further insights into factors that may be associated with increased device usage. In the follow-up, consistent long-term users were more likely to report that the device was easy to use. Although ease of use was a focus throughout product development and was reported at a relatively high level by all participants in the study (>80% overall), the results suggest that this is an issue that requires ongoing assessment in real world use. More detailed questions on ease of use suggested that less consistent users of the device may have had more difficulty completing the scan, for example, although this factor was not an independent predictor of consistent use in adjusted models. The follow-up surveys also revealed that consistent long-term users were almost twice as likely than others to report sharing results from the device with healthcare providers, suggesting that use of the device was at least partially influenced by the perceived value of the results to help guide their medical care.

Also notable in the follow-up surveys, participants overall reported changes in several health-related behaviors during the study, including approximately one-third who made positive changes in diet or exercise. An important finding was the surprisingly large percentage of participants who considered using or did utilize their results from the device to influence health decisions despite being explicitly reminded that the results were not validated and possibly inaccurate. This is especially important in light of the number of digital health devices commercially available that bypass regulatory approval and are marketed as being for entertainment purposes.[15] The results raise the concern that as these increasing numbers of unvalidated health technologies become available, their readings will likely impact personal health decisions–rightly or wrongly.

The fact that Scanadu decided not to go forward at this time with ongoing development of the Scout due to clinical accuracy concerns that precluded its application to the Food and Drug Administration reinforces the value of regulatory oversight of medical devices purporting to provide potentially actionable information.

In addition to biases associated with using investors who may be motivated to aid in the success of the device as the study cohort, as with many real-world analyses, the current study has several other important limitations related to generalizability. The cohort included mostly middle-aged men. Compared to the US population, participants in the study were more likely to live in a household with children, and, as noted, were more educated, with more than 40% holding a graduate degree. In addition, our study participants were highly educated relative to the general population, with 78% having at least an undergraduate degree, and over 40% an advanced degree, although they also had a history of trusting other home-based self-monitoring technologies. The high rates of medical and wellness devices in our study cohort were also remarkable, with almost 60% of participant households having a blood pressure monitor, for example.

Thus, from the sparse literature currently available on real-world use of digital health technologies to date, it is apparent that there is much to learn regarding user engagement. It remains to be seen whether increasing wearability and the automatic nature of device use will change the engagement equation and by how much. The value of incorporating behavioral economic principles and other motivational tools such as game elements into digital health technologies also requires further study.[16] Regardless, for health behavior change to result, merely making data visible to patients–sometimes considered “engagement” by digital health companies–will not likely be enough.[17] Development of engagement strategies, some drawn from other fields and some unique to digital medicine, will continue to be an important challenge to realizing the full health-promoting potential of this technological revolution.

## Supporting information

S1 Table Baseline characteristics not reported in manuscript Table 1. *Chi square test
(DOCX)

S2 Table Patterns of device use by number of weeks in the study. *Chi square test
(DOCX)

S3 Table Logistic regression results for association of variables with consistent long-term use of the device. *Results for model that includes all variables listed with consistent long-term use of the device (yes/no) as the outcome.
(DOCX)

S4 Table Attitudes and behaviors of participants answering surveys at 12 months by amount of device use not reported in manuscript Table 4. (DOCX)

S5 Table Attitudes and behaviors of participants answering surveys at all time points (n = 706). (DOCX)

S1 File Study data file. (XLSX)
