# Supplementary material for: Real world usage characteristics of a novel mobile health self-monitoring device: Results from the Scanadu Consumer Health Outcomes (SCOUT) Study
Source: PLoS One. 2019 Apr 16;14(4):e0215468. doi: 10.1371/journal.pone.0215468 (PMC6467418; doi:10.1371/journal.pone.0215468)
Supplement: S1 Table — *Chi square test (DOCX) [file pone.0215468.s001.docx]

S1 Table. Baseline Characteristics Not Reported in Manuscript Table 1.

| **Characteristic** | **Overall (n=3872)** | **Men (n=3249)** | **Women (n=623)** | **p*** |
| --- | --- | --- | --- | --- |
|  | **n (%)** | **n (%)** | **n (%)** |  |
| **Primary Source of Information for Managing Health** |  |  |  |  |
| Family and Friends | 180 (4.7) | 165 (5.1) | 15 (2.4) | <.0001 |
| Nurse Hotline | 7 (0.2) | 6 (0.2) | 1 (0.2) |  |
| Primary Care Provider | 1490 (38.6) | 1260 (38.9) | 230 (37.2) |  |
| Search (Google, Bing…) | 1156 (30.0) | 1004 (31.0) | 152 (24.6) |  |
| WebMD & Mayo Clinic | 198 (5.1) | 161 (5.0) | 37 (6.0) |  |
| Other | 247 (6.4) | 181 (5.6) | 66 (10.7) |  |
|  |  |  |  |  |
| **Native Language English?** | 3505 (90.5) | 2928 (90.1) | 577 (92.6) | 0.05 |
|  |  |  |  |  |
| **Share Scanadu Scout with Physician?** |  |  |  |  |
| Yes | 875 (22.6) | 760 (23.4) | 115 (18.5) | 0.02 |
| No | 432 (11.2) | 363 (11.2) | 69 (11.1) |  |
| Maybe | 2565 (66.2) | 2126 (65.4) | 439 (70.5) |  |
|  |  |  |  |  |
| **How would you share with Physician?** |  |  |  |  |
| Routine Check-up | 2147 (62.3) | 1807 (62.65) | 340 (61.3) | 0.02 |
| Medical Check-up due to abnormalities | 522 (15.2) | 424 (14.7) | 98 (17.7) |  |
| Email/Electronically Periodically | 334 (9.7) | 2967 (10.3) | 38 (6.9) |  |
| Email/Electronically due to abnormalities | 217 (6.3) | 183 (6.3) | 34 (6.1) |  |
| Other | 221 (6.4) | 176 (6.1) | 45 (8.1) |  |
|  |  |  |  |  |
| **Technology Utilization** |  |  |  |  |
| **Own Medical Device** | 2705 (70.0) | 2279 (70.3) | 426 (68.6) | 0.42 |
|  |  |  |  |  |
| **Type of Medical Device Owned** |  |  |  |  |
| Thermometer | 3099 (80.0) | 2579 (79.4) | 520 (83.5) | 0.02 |
| Scale | 3342 (86.3) | 2805 (86.3) | 537 (86.2) | 0.95 |
| BP Monitor | 2035 (52.6) | 1726 (53.1) | 309 (49.6) | 0.11 |
| Heart Rate Monitor | 1414 (36.5) | 1235 (37.9) | 179 (28.7) | <.0001 |
| Glucose Monitor | 502 (12.9) | 418 (12.8) | 84 (12.5) | 0.67 |
| Pulse Oximeter | 551 (14.2) | 477 (14.6) | 74 (11.9) | 0.07 |
| Asthma Inhaler | 291 (7.5) | 204 (6.3) | 87 (13.9) | <.0001 |
| CPAP | 350 (9.0) | 308 (9.5) | 42 (6.7) | 0.03 |
| None | 106 (2.7) | 82 (2.5) | 24 (3.9) | 0.06 |
|  |  |  |  |  |
| **When do you utilize?** |  |  |  |  |
| Wake | 1657 (42.7) | 1402 (43.1) | 255 (40.9) | 0.31 |
| Meals | 154 (4.0) | 137 (4.2) | 17 (2.7) | 0.08 |
| Meds | 63 (1.6) | 49 (1.5) | 14 (2.2) | 0.18 |
| Symptoms | 988 (25.5) | 779 (23.9) | 209 (33.5) | <.0001 |
| Exercise | 544 (14.0) | 482 (14.8) | 62 (9.9) | 0.001 |
| Random | 1755 (45.2) | 1489 (45.8) | 266 (42.6) | 0.15 |
| No use | 527 (13.3) | 433 (13.3) | 84 (13.5) | 0.91 |
|  |  |  |  |  |
|  |  |  |  |  |
|  |  |  |  |  |
|  |  |  |  |  |
|  |  |  |  |  |
| **Characteristic** | **Overall (n=3872)** | **Men (n=3249)** | **Women (n=623)** | **p*** |
|  | **n (%)** | **n (%)** | **n (%)** |  |
| **What would you do with the information?** |  |  |  |  |
| Share with family | 1989 (51.4) | 1715 (52.8) | 274 (43.9) | <.0001 |
| Document | 2977 (76.9) | 2500 (77.0) | 477 (76.6) | 0.85 |
| Take Care of Someone else | 843 (21.8) | 689 (21.2) | 154 (24.7) | 0.05 |
| Share it with physician | 2388 (61.6) | 1994 (61.3) | 394 (63.1) | 0.38 |
| Unplanned Visits | 2001 (51.7) | 1663 (51.2) | 338 (54.3) | 0.15 |
| Set Alerts | 475 (12.3) | 398 (12.2) | 77 (12.3) | 0.94 |
| Auto Send | 497 (12.8) | 429 (13.2) | 68 (10.9) | 0.12 |
| Do not use | 210 (5.5) | 171 (5.3) | 39 (6.3) | 0.33 |
|  |  |  |  |  |
| **Expect to use mobile health technology devices?** |  |  |  | <.0001 |
| At home | 2959 (76.6) | 2515 (77.5) | 444 (71.8) |  |
| On the go | 743 (19.2) | 618 (19.0) | 125 (20.2) |  |
| Other | 162 (4.2) | 112 (3.5) | 50 (8.1) |  |
|  |  |  |  |  |
| **Which device do you use?** |  |  |  |  |
| iPhone 4 | 62 (1.6) | 49 (1.5) | 13 (2.1) | 0.29 |
| iPhone 4s | 170 (4.4) | 138 (4.2) | 32 (5.1) | 0.32 |
| iPhone 5 | 326 (8.4) | 255 (7.8) | 71 (11.4) | 0.004 |
| iPhone 5s | 635 (16.2) | 523 (16.1) | 112 (18.0) | 0.24 |
| iPhone 6 | 1188 (30.6) | 1031 (31.7) | 157 (25.2) | 0.001 |
| iPhone 6 plus | 705 (18.2) | 630 (19.4) | 75 (12.0) | <.0001 |
| Samsung Galaxy S3 | 97 (2.5) | 80 (2.5) | 17 (2.7) | 0.70 |
| Samsung Galaxy S4 | 194 (5.0) | 154 (4.7) | 40 (6.4) | 0.08 |
| Samsung Galaxy 5 | 188 (4.9) | 151 (4.6) | 37 (5.9) | 0.17 |
| HTC One | 120 (3.1) | 107 (3.3) | 13 (2.1) | 0.11 |
| Google Nexus 4 | 93 (2.4) | 90 (2.8) | 3 (0.5) | 0.0006 |
| Nokia Lumina 920 | 32 (0.8) | 28 (0.9) | 4 (0.6) | 0.58 |
| None | 106 (2.7) | 82 (2.5) | 24 (3.9) | 0.06 |
|  |  |  |  |  |
| **Use Health Apps?** |  |  |  | 0.09 |
| No | 1357 (35.0) | 1112 (34.2) | 245 (39.3) |  |
| Yes; Less than 5 | 2212 (57.2) | 1877 (57.8) | 335 (53.9) |  |
| Yes; 5-10 | 265 (6.9) | 226 (7.0) | 39 (6.3) |  |
| Yes; Over 10 | 38 (1.0) | 34 (1.0) | 4 (0.6) |  |
|  |  |  |  |  |
|  |  |  |  |  |
| **H-R QOL/SF-12 Questions** |  |  |  |  |
| **How is your health?** |  |  |  | 0.43 |
| Poor | 42 (1.1) | 33 (1.0) | 9 (1.5) |  |
| Fair | 336 (8.7) | 275 (8.5) | 61 (10.1) |  |
| Good | 1445 (37.5) | 1215 (37.6) | 230 (37.3) |  |
| Very Good | 1482 (38.5) | 1242 (38.4) | 240 (38.9) |  |
| Excellent | 545 (14.1) | 469 (14.5) | 76 (12.3) |  |
|  |  |  |  |  |
| **Limitation of Climbing several flights of stairs** |  |  |  | <.0001 |
| No, not limited at all | 2881 (74.8) | 2507 (77.5) | 374 (60.6) |  |
| Yes, limited a little | 743 (19.3) | 573 (17.8) | 170 (27.6) |  |
| Yes, limited a lot | 226 (5.9) | 154 (4.8) | 72 (11.8) |  |
| **Characteristic** | **Overall (n=3872)** | **Men (n=3249)** | **Women (n=623)** | **p*** |
|  | **n (%)** | **n (%)** | **n (%)** |  |
|  |  |  |  |  |
| **Felt calm and peaceful past 4 weeks?** |  |  |  | <.0001 |
| None of the time | 18 (0.5) | 14 (0.4) | 4 (0.7) |  |
| A little of the time | 240 (6.2) | 180 (5.6) | 60 (9.7) |  |
| Some of the time | 6879 (17.9) | 556 (17.2) | 131 (21.4) |  |
| A good bit of the time | 1004 (26.1) | 834 (25.8) | 170 (27.6) |  |
| Most of the time | 1705 (44.3) | 14636 (45.3) | 242 (39.2) |  |
| All of the time | 196 (5.1) | 187 (5.8) | 9 (1.5) |  |
|  |  |  |  |  |
| **Did you have a lot of energy?** |  |  |  | <.0001 |
| None of the time | 61 (1.6) | 43 (1.3) | 18 (2.9) |  |
| A little of the time | 279 (7.3) | 204 (6.3) | 75 (12.3) |  |
| Some of the time | 896 (23.3) | 733 (22.7) | 163 (26.4) |  |
| A good bit of the time | 994 (25.8) | 835 (25.8) | 159 (25.8) |  |
| Most of the time | 1391 (36.2) | 1209 (37.4) | 182 (29.5) |  |
| All of the time | 229 (5.9) | 210 (6.5) | 19 (3.1) |  |
|  |  |  |  |  |
|  |  |  |  |  |
| **Use of Innovations** |  |  |  |  |
| **Would you find out more information about a new medical device** |  |  |  | 0.005 |
| Strongly Disagree | 16 (0.4) | 14 (0.4) | 2 (0.3) |  |
| Disagree | 60 (1.6) | 43 (1.3) | 17 (2.9) |  |
| Neutral | 662 (17.3) | 540 (16.8) | 122 (19.8) |  |
| Agree | 1974 (51.4) | 1692 (52.5) | 282 (45.8) |  |
| Strongly agree | 1128 (29.3) | 937 (29.0) | 191 (31.1) |  |
|  |  |  |  |  |
| **Hesitant to undergo new medical devices** |  |  |  | 0.31 |
| Strongly Disagree | 829 (21.6) | 714 (22.1) | 115 (18.7) |  |
| Disagree | 1855 (48.2) | 1552 (48.0) | 303 (49.3) |  |
| Neutral | 923 (24.0) | 770 (23.8) | 153 (24.9) |  |
| Agree | 200 (5.2) | 165 (5.1) | 35 (5.7) |  |
| Strongly agree | 40 (1.0) | 31 (1.0) | 9 (1.5) |  |
|  |  |  |  |  |
| **Use of Medical Devices** |  |  |  |  |
| **Mobile devices are consistent to with my health approach** |  |  |  | 0.67 |
| Strongly Disagree | 10 (0.3) | 9 (0.3) | 1 (0.2) |  |
| Disagree | 34 (0.9) | 31 (1.0) | 3 (0.5) |  |
| Neutral | 443 (11.5) | 378 (11.7) | 65 (10.6) |  |
| Agree | 1876 (48.8) | 1571 (48.7) | 305 (49.8) |  |
| Strongly agree | 1477 (38.5) | 1237 (38.4) | 240 (16.2) |  |
|  |  |  |  |  |
| **Devices are hard to understand** |  |  |  | 0.49 |
| Strongly Disagree | 1153 (30.0) | 951 (29.4) | 202 (32.9) |  |
| Disagree | 1876 (48.9) | 1586 (19.2) | 290 (47.3) |  |
| Neutral | 614 (16.0) | 520 (16.2) | 94 (15.3) |  |
| Agree | 153 (4.0) | 130 (4.0) | 23 (3.7) |  |
| Strongly agree | 44 (1.1) | 39 (1.2) | 5 (0.8) |  |
|  |  |  |  |  |
|  |  |  |  |  |
| **Characteristic** | **Overall (n=3872)** | **Men (n=3249)** | **Women (n=623)** | **p*** |
|  | **n (%)** | **n (%)** | **n (%)** |  |
|  |  |  |  |  |
| **Using the device will help me stay healthier** |  |  |  | 0.29 |
| Strongly Disagree | 20 (0.5) | 17 (0.5) | 3 (0.5) |  |
| Disagree | 151 (3.9) | 119 (3.7) | 32 (5.2) |  |
| Neutral | 1002 (26.1) | 852 (26.4) | 150 (24.4) |  |
| Agree | 1745 (45.4) | 1474 (45.6) | 271 (44.1) |  |
| Strongly agree | 929 (24.2) | 770 (23.8) | 159 (25.9) |  |
