# Supplementary material for: Real world usage characteristics of a novel mobile health self-monitoring device: Results from the Scanadu Consumer Health Outcomes (SCOUT) Study
Source: PLoS One. 2019 Apr 16;14(4):e0215468. doi: 10.1371/journal.pone.0215468 (PMC6467418; doi:10.1371/journal.pone.0215468)
Supplement: S2 Table — *Chi square test (DOCX) [file pone.0215468.s002.docx]

S2 Table. Patterns of device use by number of weeks in the study

|  | **Total Number of Weeks in the Study** | | | | |
| --- | --- | --- | --- | --- | --- |
|  | **1 week (n=508)** | **2-4 weeks**  **(n=332)** | **5-12 weeks**  **(n=716)** | **13-26 weeks**  **(n=404)** | **>26 weeks**  **(n=1513)** |
| **Average uses per week** | **n (%)** | **n (%)** | **n (%)** | **n (%)** | **n (%)** |
| ≤1 | 333 (65.6) | 82 (24.7) | 312 (43.6) | 239 (59.2) | 887 (58.6) |
| >1 and ≤2 | 90 (17.7) | 112 (33.7) | 208 (29.1) | 93 (23.0) | 349 (23.1) |
| >2 and ≤3 | 32 (6.3) | 50 (15.1) | 89 (12.4) | 30 (7.4) | 113 (7.5) |
| >3 and ≤7 | 41 (8.1) | 69 (20.8) | 92 (12.8) | 34 (8.4) | 128 (8.5) |
| >7 | 12 (2.4) | 19 (5.7) | 15 (2.1) | 8 (2.0) | 36 (2.4) |
|  |  |  |  |  |  |
|  | **Mean (SD)** | **Mean (SD)** | **Mean (SD)** | **Mean (SD)** | **Mean (SD)** |
| **Average percent of weeks with at least 1 use** | 100% | 86.5% (17.9%) | 53.4% (22.8%) | 38.6% (20.9%) | 36.2% (22.8%) |
