# Supplementary material for: Real world usage characteristics of a novel mobile health self-monitoring device: Results from the Scanadu Consumer Health Outcomes (SCOUT) Study
Source: PLoS One. 2019 Apr 16;14(4):e0215468. doi: 10.1371/journal.pone.0215468 (PMC6467418; doi:10.1371/journal.pone.0215468)
Supplement: S3 Table — *Results for model that includes all variables listed with consistent long-term use of the device (yes/no) as the outcome. (DOCX) [file pone.0215468.s003.docx]

S3 Table. Multivariable Logistic Regression Results for Association of Variables with Consistent Long-term Use of the Device at Baseline and Among Subset Answering Survey at 12 Months*

|  | **Baseline (n=3473)** | | **12 Month Survey (n=1222)** | |
| --- | --- | --- | --- | --- |
| **Variable** | **Odds Ratio (95% Confidence Interval)** | **p** | **Odds Ratio (95% Confidence Interval)** | **p** |
| **Baseline Characteristics** |  |  |  |  |
| **Age (10 year interval)** | 1.8 (1.5, 2.2) | <.001 | 1.7 (1.3, 2.1) | <.001 |
| **Household without children** | 2.2 (1.2, 4.3) | .01 | 1.8 (0.8, 3.8) | 0.14 |
| **Use other medical devices at least once daily** | 3.4 (1.5, 6.9) | .002 | 2.5 (1.0, 6.3) | 0.05 |
| **At least 1 current medical problem** | 0.9 (0.5, 1.4) | .56 | 1.3 (0.7, 2.3) | 0.45 |
| **Follow-up Survey Characteristics** |  |  |  |  |
| **Shared results with physician or health care provider** |  |  | 1.8 (1.0, 3.2) | 0.05 |
| **Agree that device was easy to use** |  |  | 3.1 (1.1, 8.8) | 0.04 |

*Results for model that includes all variables listed with consistent long-term use of the device (yes/no) as the outcome.
