# Supplementary material for: Real world usage characteristics of a novel mobile health self-monitoring device: Results from the Scanadu Consumer Health Outcomes (SCOUT) Study
Source: PLoS One. 2019 Apr 16;14(4):e0215468. doi: 10.1371/journal.pone.0215468 (PMC6467418; doi:10.1371/journal.pone.0215468)
Supplement: S4 Table — (DOCX) [file pone.0215468.s004.docx]

S4 Table. Attitudes and behaviors of participants answering surveys at 12 months by amount of device use Not Reported in Manuscript Table 4.

| **Question** | **Consistent Long-term Users**  **n=70** | **All others**  **n=1152** | **p*** |
| --- | --- | --- | --- |
| **Agree with the following:** |  |  |  |
| App instructions easy to understand | 66 (97.1) | 1055 (95.1) | 0.45 |
|  |  |  |  |
| Online instructions easy to understand | 67 (94.0) | 1053 (95.9) | 0.46 |
|  |  |  |  |
| Able to turn on device in preparation for scan | 67 (98.5) | 1089 (98.3) | 0.88 |
|  |  |  |  |
| Using the device is enjoyable | 63 (92.7) | 937 (84.9) | 0.08 |
|  |  |  |  |
| The device does not distract me from my work | 64 (94.1) | 1026 (93.3) | 0.79 |
|  |  |  |  |
| The device does not distract me from my household chores | 63 (94.0) | 1034 (93.3) | 0.82 |
|  |  |  |  |
| The device is comfortable to wear/carry around | 62 (92.5) | 937 (84.7) | 0.08 |
|  |  |  |  |
| **H-R QOL/SF-12 Questions** |  |  |  |
| **How is your health?** |  |  |  |
| Poor | 1 (1.4) | 18 (1.6) | 0.82 |
| Fair | 6 (8.6) | 122 (10.6) |  |
| Good | 25 (35.7) | 394 (34.2) |  |
| Very Good | 26 (37.1) | 472 (41.0) |  |
| Excellent | 12 (17.1) | 146 (12.7) |  |
|  |  |  |  |
| **Limitation of Climbing several flights of stairs** |  |  |  |
| Yes | 24 (34.3) | 313 (27.2) | 0.20 |
| No | 46 (65.7) | 839 (72.8) |  |
|  |  |  |  |
| **Felt calm and peaceful past 4 weeks**? |  |  |  |
| None of the time | 0 | 6 (0.5) | 0.003 |
| A little of the time | 0 | 102 (8.9) |  |
| Some of the time | 13 (18.6) | 233 (20.2) |  |
| A good bit of the time | 18 (25.7) | 304 (26.4) |  |
| Most of the time | 31 (44.3) | 468 (40.6) |  |
| All of the time | 8 (11.4) | 39 (3.4) |  |
|  |  |  |  |
| **Did you have a lot of energy?** |  |  |  |
| None of the time | 0 | 17 (1.5) | 0.07 |
| A little of the time | 3 (4.3) | 128 (11.1) |  |
| Some of the time | 16 (22.9) | 267 (23.2) |  |
| A good bit of the time | 23 (32.9) | 288 (25.0) |  |
| Most of the time | 22 (31.4) | 411 (35.7) |  |
| All of the time | 6 (8.6) | 41 (3.6) |  |
