# Supplementary material for: Real world usage characteristics of a novel mobile health self-monitoring device: Results from the Scanadu Consumer Health Outcomes (SCOUT) Study
Source: PLoS One. 2019 Apr 16;14(4):e0215468. doi: 10.1371/journal.pone.0215468 (PMC6467418; doi:10.1371/journal.pone.0215468)
Supplement: S5 Table — (DOCX) [file pone.0215468.s005.docx]

S5 Table. Attitudes and behaviors of participants answering surveys at all time points (n=706)

| **Characteristic** | **3 months** | **12 months** | **18 months** |
| --- | --- | --- | --- |
| Thought about sharing results with doctor? | 432 (61.5) | 470 (66.8) | 425 (60.7) |
|  |  |  |  |
| Thought about using for decisions on medications? | 135 (19.2) | 204 (29.0) | 190 (27.1) |
|  |  |  |  |
| Which of the following have you made changes to? |  |  |  |
| 0 = none | 596 (84.9) | 525 (74.5) | 513 (73.3) |
| 1 = non-prescription meds | 13 (1.7) | 25 (3.6) | 27 (3.9) |
| 2 = Prescription meds | 39 (5.6) | 58 (8.4) | 66 (9.4) |
| 3 = Supplements | 54 (7.8) | 96 (13.5) | 94 (13.4) |
|  |  |  |  |
| Consult with a healthcare provider about med/supplement change? | 52/107 (48.6) | 76/179 (57.5) | 89/187 (52.4) |
|  |  |  |  |
| Since using the Scanadu Scout™, how many times have you seen a medical doctor? |  |  |  |
| 0 | 411 (58.7) | 110 (15.7) | 92 (13.2) |
| 1-2 | 250 (35.7) | 338 (47.8) | 295 (42.3) |
| 3 or more | 39 (5.6) | 257 (36.2) | 311 (44.6) |
|  |  |  |  |
| Since using the Scanadu Scout™, how many times have you seen a health care professional other than a medical doctor? |  |  |  |
| 0 | 558 (79.7) | 413 (58.6) | 398 (57.0) |
| 1-2 | 116 (16.6) | 187 (26.7) | 196 (28.1) |
| 3 or more | 26 (3.7) | 102 (14.7) | 104 (14.9) |
|  |  |  |  |
| Did you share your Scanadu Scout™ results with your physician or healthcare provider? | 137 (14.8) | 224 (32.0) | 214 (30.7) |
|  |  |  |  |
| Since using the Scanadu Scout™, have you made any changes to your typical diet? | 102 (14.6) | 196 (27.9) | 206 (29.5) |
|  |  |  |  |
| If yes, please specify how your diet has changed |  |  |  |
| Decreased healthy foods significantly | 0 | 2 (1.0) | 2 (1.0) |
| Decreased healthy foods slightly | 9 (8.8) | 6 (3.1) | 1 (0.5) |
| Increased healthy foods slightly | 65 (63.7) | 122 (63.2) | 123 (60.6) |
| Increased healthy foods significantly | 28 (27.5) | 63 (32.67) | 77 (37.9) |
|  |  |  |  |
| Since using the Scanadu Scout™, have you made any changes to your exercise routine? | 167 (23.9) | 238 (33.9) | 252 (36.3) |
|  |  |  |  |
| If yes, please specify how your exercise has changed |  |  |  |
| Decreased exercise significantly | 4 (2.4) | 7 (2.9) | 7 (2.8) |
| Decreased exercise slightly | 9 (5.3) | 6 (2.5) | 14 (5.6) |
| Increased exercise slightly | 116 (68.2) | 161 (67.7) | 156 (62.2) |
| Increased exercise significantly | 41 (24.1) | 64 (26.9) | 74 (29.5) |
|  |  |  |  |
| **Characteristic** | **3 months** | **12 months** | **18 months** |
| Would you recommend the Scanadu Scout™ to family members or friends? | 580 (83.3) | 576 (82.6) | 539 (78.5) |
|  |  |  |  |
| Overall, how satisfied are you with your Scanadu Scout™? |  |  |  |
| Very dissatisfied | 20 (2.4) | 23 (3.3) | 25 (3.7) |
| Dissatisfied | 72 (10.5) | 62 (9.0) | 82 (12.0) |
| Satisfied | 400 (58.4) | 358 (51.9) | 322 (47.3) |
| Very Satisfied | 193 (28.2) | 247 (35.8) | 252 (37.0) |
|  |  |  |  |
| Overall, how easy/difficult was it to use your Scanadu Scout™? |  |  |  |
| Very dissatisfied | 20 (3.1) | 37 (5.4) | 31 (4.6) |
| Dissatisfied | 121 (17.6) | 73 (10.6) | 88 (13.0) |
| Satisfied | 363 (52.8) | 302 (43.8) | 284 (41.8) |
| Very Satisfied | 182 (26.5) | 278 (40.3) | 276 (40.7) |
|  |  |  |  |
| Overall, the App Instructions for Use and guidelines were easy to understand and follow. |  |  |  |
| Strongly disagree | 3 (0.4) | 8 (1.2) | 3 (0.4) |
| Disagree | 30 (4.4) | 232 (3.3) | 32 (4.7) |
| Agree | 272 (39.8) | 223 (32.3) | 232 (34.1) |
| Strongly agree | 378 (55.3) | 436 (63.2) | 414 (60.8) |
|  |  |  |  |
| Overall, the online Instructions for Use were easy to understand and follow. |  |  |  |
| Strongly disagree | 3 (0.4) | 6 (0.9) | 1 (0.2) |
| Disagree | 24 (3.5) | 21 (3.1) | 27 (4.0) |
| Agree | 294 (43.2) | 240 (35.1) | 244 (36.1) |
| Strongly agree | 360 (52.9) | 417 (61.0) | 4014 (59.8) |
|  |  |  |  |
| Was able to open and successfully connect the Scanadu Scout™ to the app. |  |  |  |
| Strongly disagree | 12 (1.8) | 9 (1.3) | 5 (0.7) |
| Disagree | 31 (4.5) | 26 (3.8) | 29 (4.3) |
| Agree | 198 (28.8) | 149 (21.6) | 173 (25.4) |
| Strongly agree | 446 (64.9) | 505 (73.3) | 474 (69.6) |
|  |  |  |  |
| Was able to turn on the device in preparation for a scan |  |  |  |
| Strongly disagree | 5 (0.7) | 2 (0.3) | 0 |
| Disagree | 8 (1.2) | 11 (1.6) | 4 (0.6) |
| Agree | 167 (24.3) | 108 (15.7) | 145 (21.3) |
| Strongly agree | 506 (73.8) | 569 (82.5) | 531 (78.1) |
|  |  |  |  |
| Was able to complete a successful scan and obtain the measurements. |  |  |  |
| Strongly disagree | 33 (4.8) | 35 (5.1) | 37 (5.4) |
| Disagree | 128 (18.6) | 91 (13.2) | 83 (12.2) |
| Agree | 306 (44.5) | 270 (39.1) | 282 (41.5) |
| Strongly agree | 220 (32.0) | 294 (42.6) | 278 (40.9) |
|  |  |  |  |
| **Characteristic** | **3 months** | **12 months** | **18 months** |
| Overall, my Scanadu Scout™ interrupted my daily activities. |  |  |  |
| Strongly disagree | 134 (20.7) | 348 (50.4) | 326 (48.0) |
| Disagree | 61 (9.4) | 233 (33.8) | 211 (31.1) |
| Agree | 255 (39.3) | 60 (8.7) | 84 (12.4) |
| Strongly agree | 199 (30.7) | 49 (7.1) | 58 (8.5) |
|  |  |  |  |
| Overall, using my Scanadu Scout™ was an enjoyable experience. |  |  |  |
| Strongly disagree | 18 (2.6) | 23 (3.3) | 29 (4.3) |
| Disagree | 57 (8.3) | 63 (9.1) | 73 (10.7) |
| Agree | 316 (46.2) | 297 (43.1) | 294 (43.2) |
| Strongly agree | 293 (42.8) | 306 (44.1) | 285 (41.9) |
|  |  |  |  |
| Overall, using my Scanadu Scout™ required too much of my time. |  |  |  |
| Strongly disagree | 155 (23.6) | 347 (50.4) | 311 (45.8) |
| Disagree | 119 (18.1) | 218 (31.67) | 231 (34.0) |
| Agree | 220 (33.4) | 89 (12.9) | 90 (13.3) |
| Strongly agree | 164 (24.9) | 35 (5.1) | 47 (6.9) |
|  |  |  |  |
| Overall, my Scanadu Scout™ was distracting |  |  |  |
| Strongly disagree | 161 (24.7) | 406 (59.4) | 359 (53.4) |
| Disagree | 103 (15.8) | 216 (31.6) | 232 (34.5) |
| Agree | 216 (33.1) | 43 (6.3) | 53 (7.9) |
| Strongly agree | 173 (26.5) | 19 (2.8) | 28 (4.2) |
|  |  |  |  |
| Overall, my Scanadu Scout™ distracted me from my work. |  |  |  |
| Strongly disagree | 172 (26.3) | 456 (66.7) | 417 (61.77) |
| Disagree | 92 (14.1) | 193 (28.2) | 206 (30.5) |
| Agree | 202 (30.8) | 18 (2.6) | 30 (4.4) |
| Strongly agree | 189 (28.9) | 17 (2.5) | 23 (3.4) |
|  |  |  |  |
| Overall, my Scanadu Scout™ distracted me from my household chores. |  |  |  |
| Strongly disagree | 172 (26.3) | 457 (66.3) | 421 (61.7) |
| Disagree | 91 (13.9) | 196 (28.5) | 202 (30.5) |
| Agree | 202 (30.8) | 16 (2.3) | 30 (4.4) |
| Strongly agree | 190 (29.0) | 20 (2.9) | 23 (3.4) |
|  |  |  |  |
| Overall, my Scanadu Scout™ was uncomfortable to wear/carry around. |  |  |  |
| Strongly disagree | 159 (24.3) | 399 (58.1) | 351 (52.2) |
| Disagree | 93 (14.2) | 202 (29.4) | 219 (32.54) |
| Agree | 216 (33.0) | 55 (8.0) | 72 (10.7) |
| Strongly agree | 187 (28.6) | 31 (4.5) | 31 (4.6) |
|  |  |  |  |
| Were you able to easily understand and follow the instructions for use of the device? | 671 (97.8) | 679 (98.4) | 661 (97.2) |
|  |  |  |  |
|  |  |  |  |
| **Characteristic** | **3 months** | **12 months** | **18 months** |
| I would be willing to participate in a study testing my Scanadu Scout™ again? | 659 (95.9) | 621 (90.1) | 588 (86.2) |
|  |  |  |  |
| **H-R QOL/SF-12 Questions** |  |  |  |
| **Limitation of Moderate Activities** |  |  |  |
| Yes | 143 (20.3) | 151 (21.3) | 148 (21.1) |
|  |  |  |  |
| **How is your health?** |  |  |  |
| Poor | 10 (1.4) | 9 (1.3) | 10 (1.4) |
| Fair | 72 (10.2) | 77 (10.9) | 71 (10.1) |
| Good | 245 (34.9) | 240 (34.0) | 250 (35.7) |
| Very Good | 280 (39.8) | 291 (41.3) | 274 (39.1) |
| Excellent | 96 (13.7) | 88 (12.5) | 96 (13.7) |
|  |  |  |  |
| **Limitation of Climbing several flights of stairs** |  |  |  |
| Yes | 202 (28.7) | 201 (28.7) | 199 (28.4) |
|  |  |  |  |
| **Felt calm and peaceful past 4 weeks?** |  |  |  |
| None of the time | 3 (0.4) | 3 (0.4) | 5 (0.7) |
| A little of the time | 48 (6.8) | 58 (8.2) | 59 (8.4) |
| Some of the time | 128 (18.2) | 140 (19.9) | 129 (18.4) |
| A good bit of the time | 198 (28.2) | 178 (25.3) | 168 (24.0) |
| Most of the time | 311 (44.2) | 295 (41.8) | 314 (77.8) |
| All of the time | 15 (2.1) | 31 (4.4) | 26 (3.7) |
|  |  |  |  |
| **Did you have a lot of energy?** |  |  |  |
| None of the time | 13 (1.9) | 9 (1.3) | 15 (2.1) |
| A little of the time | 55 (7.8) | 76 (10.8) | 73 (10.4) |
| Some of the time | 189 (26.9) | 168 (23.8) | 172 (24.5) |
| A good bit of the time | 189 (26.9) | 173 (24.5) | 169 (24.1) |
| Most of the time | 236 (33.6) | 252 (35.7) | 243 (34.7) |
| All of the time | 21 (3.0) | 27 (3.8) | 29 (4.1) |
